# Supplementary material for: An open-source framework for large-scale, flexible evaluation of biomedical text mining systems
Source: J Biomed Discov Collab. 2008 Jan 29;3:1. doi: 10.1186/1747-5333-3-1 (PMC2276192; doi:10.1186/1747-5333-3-1)
Supplement: Additional file 1 — Evaluation platform source code. The additional file contains the software evaluation framework discussed in this paper. The most current release can be downloaded from SourceForge.net [1]. See the accompanying README file for instructions on installation and use. Also included in the distribution is a collection of UIMA wrappers for some commonly used BioNLP tools and annotated corpora. [file 1747-5333-3-1-S1.zip › bionlp_uima_repository-1.0.1/docs/api/src-html/edu/uchsc/ccp/uima/cc/summary/comparison/ComparatorConfigurator.html]

```
001    package edu.uchsc.ccp.uima.cc.summary.comparison;
002    
003    import java.io.IOException;
004    import java.util.ArrayList;
005    import java.util.HashMap;
006    import java.util.Iterator;
007    import java.util.List;
008    import java.util.Map;
009    
010    import org.jdom.Document;
011    import org.jdom.Element;
012    import org.jdom.JDOMException;
013    import org.jdom.input.SAXBuilder;
014    
015    import edu.uchsc.ccp.util.xml.XMLValidityChecker;
016    
017    /**
018     * This is a utility class for configuring an AnnotationComparator. Its main purpose is to read the config file for the
019     * AnnotationComparator.
020     * 
021     * The format for the config file is as follows:
022     * 
023     * <pre>
024     *    &lt;!-- Settings for a single AnnotationComparator run --&gt;
025     *    &lt;AnnotationComparatorSettings&gt;
026     *   
027     *    &lt;!-- AnnotationGroups define the annotations that will be used in the comparison. Each group          --&gt;
028     *    &lt;!-- is identified by a unique GroupID, and has annotation members defined by their                   --&gt;
029     *    &lt;!-- AnnotationSetID, AnnotatorID, and AnnotationType (which is equivalent to the ClassMention type). --&gt;
030     *    &lt;!-- Multiple AnnotationGroups are permitted. --&gt;
031     *    &lt;AnnotationGroup&gt;
032     *       &lt;GroupID&gt;[Group ID]&lt;/GroupID&gt;
033     *       &lt;AnnotationSetID&gt;[Annotation Set ID]&lt;/AnnotationSetID&gt;
034     *       &lt;AnnotatorID&gt;[Annotator ID]&lt;/AnnotatorID&gt;
035     *       &lt;!-- Multiple AnnotationTypes are permitted --&gt;
036     *       &lt;AnnotationType&gt;[Type String]&lt;/AnnotationType&gt;
037     *    &lt;/AnnotationGroup&gt;
038     *    
039     *    &lt;!?ComparisonGroups define how the annotations are compared. Currently, only pairwise comparisons are --&gt;
040     *    &lt;!-- enabled. The ComparisonGroup with GoldStandard set to true is used as the gold standard, and all --&gt;
041     *    &lt;!-- other groups are compared against it.                                                            --&gt;
042     *    &lt;ComparisonGroup&gt;
043     *       &lt;!-- If the GoldStandard tag is not present, its value is presumed to be false. --&gt;
044     *       &lt;GoldStandard&gt;[true or false]&lt;/GoldStandard&gt;
045     *       &lt;ComparisonGroupDescription&gt;[Description String]&lt;/ComparisonGroupDescription&gt;
046     *       &lt;!-- Multiple AnnotationGrouPIDs are permitted --&gt;
047     *       &lt;AnnotationGroupID&gt;[Annotation Group ID]&lt;/AnnotationGroupID&gt;
048     *    &lt;/ComparisonGroup&gt;
049     *    
050     *    &lt;/AnnotationComparatorSettings&gt;
051     * </pre>
052     * 
053     * 
054     * @author williamb
055     * 
056     */
057    
058    public class ComparatorConfigurator {
059    private final boolean DEBUG = true;
060        
061        private Map<AnnotationGroupID, AnnotationGroup> annotationGroupMap;
062    
063        private List<ComparisonGroup> comparisonGroupList;
064    
065        private final String ANNOTATION_GROUP_TAG = "AnnotationGroup";
066    
067        private final String ANNOTATION_GROUP_ID_TAG = "GroupID";
068    
069        private final String ANNOTATION_GROUP_ANNOTATORID_TAG = "AnnotatorID";
070    
071        private final String ANNOTATION_GROUP_ANNOTATIONSETID_TAG = "AnnotationSetID";
072    
073        private final String ANNOTATION_GROUP_ANNOTATIONTYPE_TAG = "AnnotationType";
074    
075        private final String COMPARISON_GROUP_TAG = "ComparisonGroup";
076    
077        private final String COMPARISON_GROUP_ISGOLDSTANDARD_TAG = "GoldStandard";
078    
079        private final String COMPARISON_GROUP_DESCRIPTION_TAG = "ComparisonGroupDescription";
080    
081        private final String COMPARISON_GROUP_ANNOTATIONGROUPMEMBER_TAG = "AnnotationGroupID";
082    
083        private boolean hasGoldGroup = false;
084        
085        public ComparatorConfigurator(String configFile) {
086            /* initialize containers for AnnotationGroups and ComparisonGroups */
087            annotationGroupMap = new HashMap<AnnotationGroupID, AnnotationGroup>();
088            comparisonGroupList = new ArrayList<ComparisonGroup>();
089    
090            /* parse the configuration file, filling the group containers as you go */
091            try {
092                parseConfigFile(configFile);
093            } catch (IOException e) {
094                System.err.println("ERROR -- ComparatorConfigurator: IOException while parsing configuration file.");
095                e.printStackTrace();
096            } catch (JDOMException e) {
097                System.err.println("ERROR -- ComparatorConfigurator: JDOMException while parsing configuration file.");
098                e.printStackTrace();
099            }
100    
101        }
102    
103        private void parseConfigFile(String configFile) throws IOException, JDOMException {
104            /* check for valid xml */
105            if (XMLValidityChecker.validateXML(configFile)) {
106    
107                SAXBuilder saxBuilder = new SAXBuilder();
108                Document document = saxBuilder.build(configFile);
109                Element rootElement = document.getRootElement();
110    
111                hasGoldGroup = false;
112                
113                Iterator childrenIterator = rootElement.getChildren().iterator();
114    
115                while (childrenIterator.hasNext()) {
116                    Element childElement = (Element) childrenIterator.next();
117                    if (childElement.getName().equals(ANNOTATION_GROUP_TAG)) {
118                        processAnnotationGroupElement(childElement);
119                    } else if (childElement.getName().equals(COMPARISON_GROUP_TAG)) {
120                        processComparisonGroupElement(childElement);
121                    } else {
122                        System.err.println("WARNING -- ComparatorConfigurator: Unexpected element encountered: " + childElement.getName());
123                    }
124                }
125    
126            } else {
127                System.err.println("ERROR -- ComparatorConfiguration: Configuration file is not well-formed XML. Please fix and try again.");
128            }
129        }
130    
131        /*
132         * Get the attributes of an AnnotationGroup and add a new AnnotationGroup object to the annotationGroupMap
133         */
134        private void processAnnotationGroupElement(Element annotationGroupElement) {
135            /* Initialize a new AnnotationGroup */
136            AnnotationGroup aGroup = new AnnotationGroup();
137    
138            try {
139                Iterator attributeIter = annotationGroupElement.getChildren().iterator();
140                while (attributeIter.hasNext()) {
141                    Object possibleElement = attributeIter.next();
142                    if (possibleElement instanceof Element) {
143                        Element attribute = (Element) possibleElement;
144                        if (attribute.getName().equals(ANNOTATION_GROUP_ID_TAG)) {
145                            aGroup.setGroupID(new AnnotationGroupID(attribute.getValue()));
146                        } else if (attribute.getName().equals(ANNOTATION_GROUP_ANNOTATIONSETID_TAG)) {
147                            aGroup.setAnnotationSetID(Integer.parseInt(attribute.getValue()));
148                        } else if (attribute.getName().equals(ANNOTATION_GROUP_ANNOTATORID_TAG)) {
149                            aGroup.setAnnotatorID(Integer.parseInt(attribute.getValue()));
150                        } else if (attribute.getName().equals(ANNOTATION_GROUP_ANNOTATIONTYPE_TAG)) {
151                            aGroup.addAnnotationType(attribute.getValue());
152                        } else {
153                            System.err.println("WARNING -- ComparatorConfigurator: Unexpected Element found in AnnotationGroup: "
154                                    + attribute.getName());
155                        }
156                    } else {
157                        System.err.println("WARNING -- ComparatorConfigurator: Expected Element in processAnnotationGroupElement but got "
158                                + possibleElement.getClass().getName());
159                    }
160                }
161    
162                /* If all fields of an annotation group have been filled, then store it in the annotationGroupMap */
163                if (aGroup.isValid()) {
164                    
165                    System.out.println("CONTAINS 0 = " + annotationGroupMap.containsKey(new AnnotationGroupID("0")));
166                    System.out.println("get 0: " + annotationGroupMap.get(new AnnotationGroupID("0")));
167                    if (annotationGroupMap.containsKey(aGroup.getGroupID())) {
168                        System.err.println("WARNING -- ComparatorConfigurator: Duplicate AnnotationGroup IDs detected. A group has been discarded.");
169                    } else {
170                        this.annotationGroupMap.put(aGroup.getGroupID(), aGroup);
171                    }
172                } else {
173                    System.err.println("WARNING -- ComparatorConfigurator: Invalid AnnotationGroup detected.");
174                }
175            } catch (NumberFormatException nfe) {
176                System.err
177                        .println("ERROR -- ComparatorConfigurator: Caught a NumberFormatException. Please check to make sure AnnotatorID and AnnotationSetID fields are integers in all AnnotationGroups.");
178            }
179        }
180    
181        /*
182         * Get the attributes of a ComparisonGroup and add a new ComparisonGroup object to the commparisonGroupList
183         */
184        private void processComparisonGroupElement(Element comparisonGroupElement) {
185            /* Initialize a new ComparisonGroup */
186            ComparisonGroup cGroup = new ComparisonGroup();
187    
188            Iterator attributeIter = comparisonGroupElement.getChildren().iterator();
189            while (attributeIter.hasNext()) {
190                Object possibleElement = attributeIter.next();
191                if (possibleElement instanceof Element) {
192                    Element attribute = (Element) possibleElement;
193                    if (attribute.getName().equals(COMPARISON_GROUP_ISGOLDSTANDARD_TAG)) {
194                        
195                        boolean isGoldGroup = Boolean.valueOf(attribute.getValue());
196                        cGroup.setGoldStandard(isGoldGroup);
197                        
198                        if (DEBUG) {
199                            System.err.println("DEBUG -- ComparatorConfigurator: isGS text = " + attribute.getValue());
200                            System.err.println("DEBUG -- ComparatorConfigurator: cGroup.isGoldStandard()=" + cGroup.isGoldStandard());
201                            //cGroup.print(System.err);
202                        }
203                        if (isGoldGroup & hasGoldGroup) {
204                            System.err.println("WARNING -- ComparatorConfigurator: Multiple Gold Standard Comparison groups found. Only one may be used.");
205                        } else if (isGoldGroup) {
206                            hasGoldGroup = true;
207                        }
208                        
209                    } else if (attribute.getName().equals(COMPARISON_GROUP_DESCRIPTION_TAG)) {
210                        cGroup.setDescription(attribute.getValue());
211                    } else if (attribute.getName().equals(COMPARISON_GROUP_ANNOTATIONGROUPMEMBER_TAG)) {
212                        cGroup.addAnnotationGroup(new AnnotationGroupID(attribute.getValue()));
213                    } else {
214                        System.err.println("WARNING -- ComparatorConfigurator: Unexpected Element found in ComparisonGroup: "
215                                + attribute.getName());
216                    }
217                } else {
218                    System.err.println("WARNING -- ComparatorConfigurator: Expected Element in processComparisonGroupElement but got "
219                            + possibleElement.getClass().getName());
220                }
221            }
222    
223            /* If all fields of an annotation group have been filled, then store it in the annotationGroupMap */
224            if (cGroup.isValid()) {
225                this.comparisonGroupList.add(cGroup);
226                if (DEBUG) {
227                    System.err.println("DEBUG -- ComparatorConfigurator: Adding cGroup to group list.");
228                    cGroup.print(System.err);
229                }
230            } else {
231                System.err.println("WARNING -- ComparatorConfigurator: Invalid ComparisonGroup detected.");
232            }
233    
234        }
235    
236        public Map<AnnotationGroupID, AnnotationGroup> getAnnotationGroupMap() {
237            return annotationGroupMap;
238        }
239    
240        public List<ComparisonGroup> getComparisonGroupList() {
241            return comparisonGroupList;
242        }
243    
244    }
```
